# Plant Litter Trait Variation Between Native and Invasive Species Across Steep Climate Gradients in the Hawaiian Islands

**DOI:** 10.1002/ece3.73030

**Published:** 2026-02-09

**Authors:** Manichanh Satdichanh, Rebecca Ostertag, William Harrigan, Mahdi Belcaid, Kasey E. Barton

**Affiliations:** ^1^ School of Life Sciences University of Hawaiʻi at Mānoa Honolulu Hawaiʻi USA; ^2^ Department of Biology University of Hawaiʻi at Hilo Hawaiʻi USA; ^3^ Hawaiʻi Institute of Marine Biology University of Hawaiʻi at Mānoa Honolulu Hawaiʻi USA; ^4^ Department of Computer Science University of Hawaiʻi at Mānoa Honolulu Hawaii USA

**Keywords:** biological invasion, decomposition, functional traits, island biodiversity, leaf economics spectrum, meta‐analysis, trait‐based ecology

## Abstract

Oceanic islands have high biodiversity, which is severely threatened by invasive species. Functional traits serve as a framework to investigate invasive‐native dynamics, but most studies investigating native‐invasive plant functional trait differences on islands focus on live foliage traits, while litter traits remain understudied. It is hypothesized that invasive species produce higher quality litter (e.g., high nutrient content, low tannins and leaf mass per area) than native species, and furthermore, that this high‐quality litter decomposes more rapidly, in turn providing a positive feedback that facilitates their expansion. To investigate native vs. invasive plant litter quality in a highly endemic island flora, we conducted a systematic review to synthesize litter trait data from Hawaiʻi. To account for the extensive heterogeneity that occurs across the Hawaiian Islands, litter trait variability was synthesized with respect to elevation and climate gradients. Litter quality varies extensively across the Hawaiian Islands in native and invasive species. Although invasive plants have higher quality litter than native species overall, species origin accounts for relatively little trait variance, and native and invasive species overlap considerably in litter multivariate trait space. Moreover, intraspecific variation exceeds interspecific variation, highlighting the important role of environmental heterogeneity for widespread species. Climate influences native and invasive litter quality in distinct ways, leading to a reversal in strategy across climate gradients. When controlling for the full direct effects of climate, native and invasive plant litter traits are not significantly different. Climate heterogeneity, more than plant species origin, plays a key role in shaping plant litter trait variation and resource‐use strategies at the landscape or archipelago scale. Litter quality could be more commonly sampled as part of the functional syndrome of plants and for a better understanding of how traits differ between native and invasive plants.

## Graphical abstract

Plant litter quality (nutrient content and toughness) varies extensively within native and invasive Hawaiian plants, with strong links to climate. Minimal differentiation between native and invasive species indicates weak support for the prediction that invasives have higher quality litter associated with fast decomposition and resource use strategies.

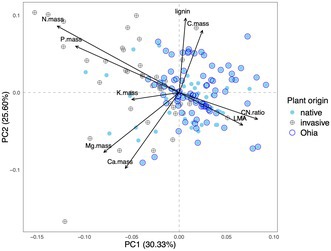

## 1 Introduction

Islands support a disproportionate extent of global biodiversity, with exceptionally high rates of endemicity on remote islands (Carlquist 1974). Island biodiversity is among the most threatened and endangered, with invasive species posing a major threat to island floras (Fernández‐Palacios et al. 2021). High propagule pressure and species interactions are broadly implicated in the success of invasive plants on islands (Daehler 2006; Russell and Kueffer 2019), but a comprehensive model for the mechanisms by which invaders displace native plants remains elusive. While competitive displacement is generally assumed to be a major driver of island plant declines (Denslow 2003), the evidence remains mixed, with relatively scant direct evidence for competition via resource‐use strategies (Barton and Fortunel 2023).

Functional traits offer a promising approach to investigate invasive‐native dynamics because of their links to fitness, mechanistic insights, and relative ease of sampling (Violle et al. 2007). It has been predicted that invasive plants with life history strategies conferring rapid growth have functional trait values related to fast resource use, and that this underlies their displacement of native plants with more conservative resource use and slow growth strategies (Pyšek and Richardson 2007). While evidence has accumulated to support this prediction on continents (Ordoñez et al. 2010; van Kleunen et al. 2010), the evidence that native island plants have more conservative traits than invasive plants is limited (Barton and Fortunel 2023), with notable exceptions from Hawaiʻi (Westerband et al. 2021) and the Canary Islands (Kühn et al. 2021).

Most studies investigating native and invasive plant functional traits on islands focus on leaf and wood traits. Litter functional traits may offer unique insights into the dual roles of invasive species as potential competitors and architects of ecosystem change as they are both effect and response traits (Lavorel and Garnier 2002). Through processes of senescence and nutrient resorption, leaf litter traits often correlate with live leaf traits (Cornwell et al. 2008). Thus, litter traits can be considered an extension of plant functional strategies, including the leaf economics spectrum (Wright et al. 2004; Santiago 2007; Lin et al. 2019). Litter quantity and quality also influence the rate of nutrient cycling (Coûteaux et al. 1995), providing a link between invasive plant establishment and their effects on ecosystem function. Invasive species are predicted to have fast resource‐use functional strategies (e.g., short‐lived leaves with high nutrient content and low toughness), corresponding to high‐quality, acquisitive litter traits (e.g., high nutrient content, low tannins and leaf mass per area) with fast decomposition rates, thereby increasing nutrient availability in the soil, which in turn establishes a positive‐feedback loop that supports the acquisitive strategy that underlies invasive plant success (Castro‐Díez et al. 2014; Lee et al. 2017; Liao et al. 2008).

Invasive species establishment and litter production have also been linked to abiotic factors such as climate and soil nutrient availability (D'Antonio et al. 2017). For example, warm and moist climates are predicted to favor fast‐growing plant species that produce large amounts of high‐quality litter annually, while cold and dry climates are predicted to promote slow‐growing plant species that produce low‐quality litter (Joswig et al. 2022; Pugnaire et al. 2019). Studies have shown that invasive plants lead to accelerated nitrogen fluxes at sites with warm and moist climates (Castro‐Díez et al. 2014), contributing to plant productivity and soil nutrient dynamics (Lee et al. 2017). However, these generalities are not absolute as litter decomposition studies have also detected similar rates for native and invasive species (Jo et al. 2016). Therefore, understanding the extent and drivers of litter trait variability between native and invasive species is essential for predicting their impacts on ecosystem functions. Although previous research conducted on islands has investigated native vs. invasive plant litter variation within sites, litter dynamics associated with plant invasion remain unclear at the landscape or archipelago scale, where climate and soil variability are extensive.

The Hawaiian Islands are uniquely positioned to be a model for characterizing litter trait variability of native vs. invasive plants. As the most remote archipelago, with exceptionally high endemism (> 90% for angiosperms) (Wagner et al. 2005), Hawaiʻi has experienced incredible rates of species introductions, and invasive plant species now outnumber native species (Brock and Daehler 2021). The Hawaiian Islands are of volcanic origin, and the current eight main islands represent an approximately 5‐million‐year chronosequence of substrate age, which along with tall mountains with steep climate gradients (Ziegler 2002), provides a model system for the study of community assembly, ecological succession, substrate development, and ecosystem function (Vitousek 2004, 1995). Across the islands, there is a substrate‐age gradient that corresponds to a shift in soil nutrients (Crews et al. 1995). The youngest soils are associated with volcanic activity, currently found on Hawaiʻi Island, and these young soils are nitrogen‐limited due to the newly deposited volcanic substrate that contains mostly “rock‐derived nutrients” such as phosphorus (P), calcium (Ca), magnesium (Mg), and potassium (K), while the oldest soils contain high nitrogen (N) levels that accumulated via biological fixation, but low levels of rock derived nutrients due to weathering (Chadwick et al. 1999), and the intermediate‐age substrates contain lower Ca, Mg, K, but higher N and P (Vitousek and Farrington 1997). While a strength of the long‐substrate age gradient extensively studied by researchers is the consistent climate across the substrates of variable ages (Crews et al. 1995; Vitousek 1995), other work in the Hawaiian Islands has shown that soil nutrients interact strongly with climate, leading to complex patterns in nutrient cycling within and across the islands (Vitousek 2004; Barton et al. 2021).

Numerous studies have documented substantial variability in litter traits among and within species in Hawaiʻi (Allison and Vitousek 2004; Hobbie 2000; Rothstein et al. 2004). While many of these studies have sampled litter across multiple sites, it is challenging to scale from a few sites to the extensive climate gradients across the archipelago. Here we synthesize this large body of litter trait data across the Hawaiian archipelago using systematic review methods and quantitative analyses in order to characterize the full extent of litter variability across the islands. We focused our synthesis on litter quality traits linked to the leaf economics spectrum and decomposition rates, including a morphological trait (leaf mass per area) and multiple traits of chemical content: lignin, carbon, nitrogen, phosphorus, calcium, magnesium, potassium (Table S1). Our primary goals are to characterize litter trait variability between native Hawaiian plants and non‐native plants established in natural habitats (i.e., invasive species) and to investigate patterns across climate gradients. We address four main questions: (1) Do litter traits covary into syndromes associated with resource economics and decomposition? (2) Do invasive plants have high‐quality litter reflective of a more acquisitive strategy with fast decomposition rates compared to native plant litter? (3) Are native‐invasive litter trait differences consistent across climate gradients? (4) What are the sources (e.g., plant species origin, plant form, intra‐ and inter‐specific variation) of litter trait variability?

## 2 Methods

### 2.1 Systematic Review Methods

Using systematic review methods, we surveyed published studies reporting litter quality trait data of plants sampled in Hawaiʻi, which ultimately yielded 35 papers published between 1990 and 2019 with suitable data (see Appendix S1 for search method details and full list of Data Sources). Published studies included in the synthesis met these inclusion criteria: (1) litter traits related to resource economics and decomposition (i.e., litter quality traits) were measured at the individual species scale (i.e., not bulk sampled or quantified at the community scale); (2) traits were measured under natural field conditions (excluding garden or greenhouse experiments, or plants grown in cultivation); (3) species were identified; and (4) data were available in tabular, graphical, or archived format. Trait means presented in graphs were estimated using WebPlot Digitizer (Version 4.8).

**FIGURE 1:**
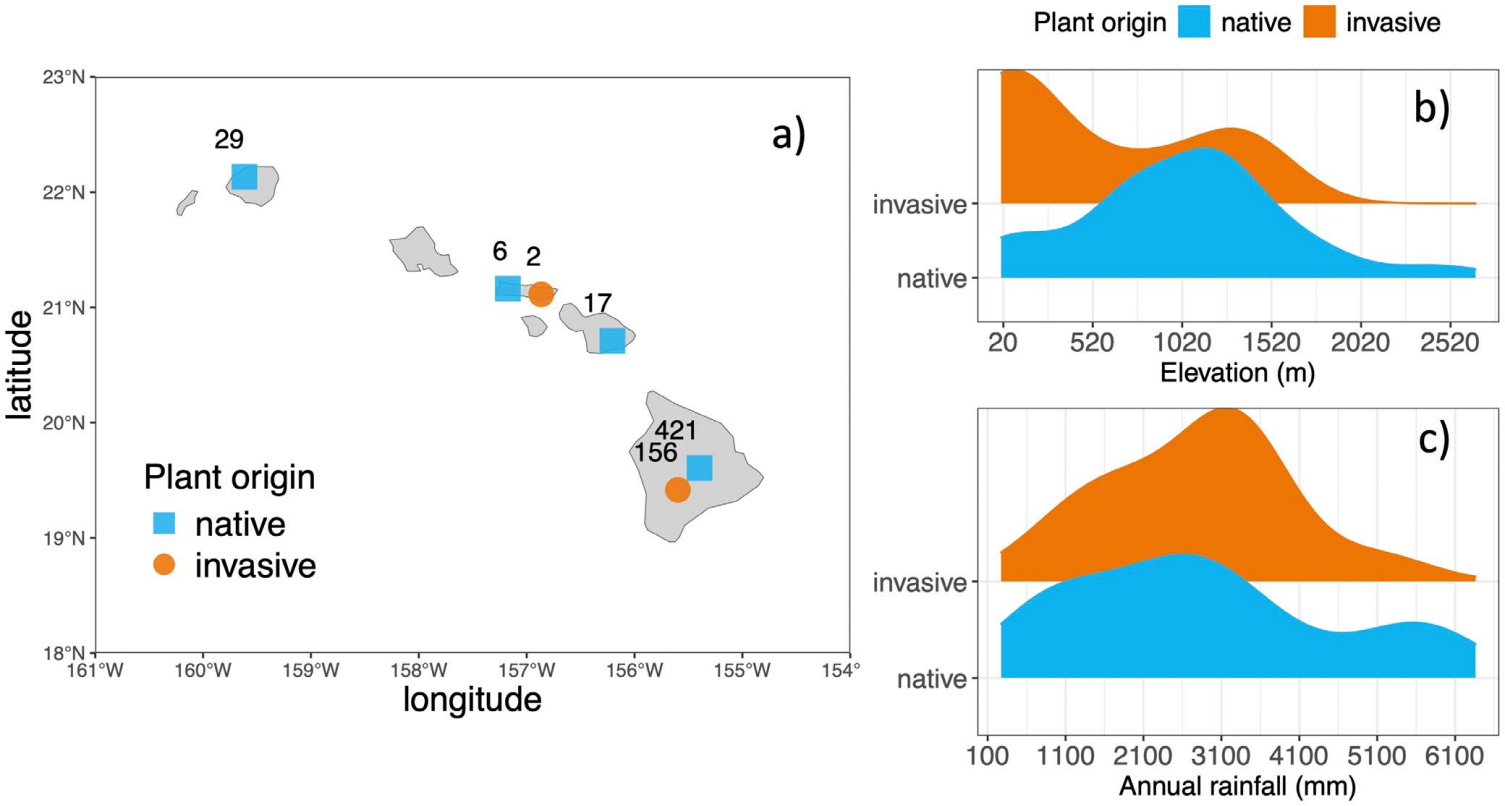
Data coverage maps for this synthesis. (a) Collection sites of litter trait data across the Hawaiian archipelago. (b) Distribution of litter trait data across elevational gradients. (c) Distribution of litter trait data across rainfall gradients. Sample sizes of litter species trait means are reported for each island with available published data. Datapoints with unknown locations are excluded (*n* = 22 for natives, *n* = 34 for invasives).

Litter trait sampling was most extensive on Hawaiʻi Island, accounting for more than 60% of the data (Figure 1). Although native species were sampled across four of the eight main Hawaiian Islands, invasive species were largely sampled only on Hawaiʻi Island, leading to an unbalanced comparison of invasive vs. native litter traits with respect to island. Notably, there were no litter trait data published from Oʻahu, emphasizing the need for future research before robust characterizations can be made across the island chronosequence.

### 2.2 Litter Traits, Plant Form, and Phylogenetic Signal

The final dataset included a total of 687 data points of litter trait means, sampled from 42 plant species (17 native and 25 invasive) belonging to 29 families (Table S2). The traits analyzed include leaf mass per area of litter (LMA; g m^−2^) and chemical content measured on a dry mass basis for: lignin (mg g^−1^), nitrogen (N.mass; mg g^−1^), phosphorus (P.mass; mg g^−1^), carbon (C.mass; mg·g^−1^), C‐to‐N ratio (C:N), potassium (K.mass; mg·g^−1^), magnesium (Mg. mass; mg g^−1^), and calcium (Ca.mass; mg g^−1^) (Table S1 for more details). The total sample sizes for each trait for native and invasive species ranged from 22 to 590 (Table S3). All data analyses were performed using R version 4.4.1 (R Core Team 2024).

Plant species names were cross referenced and standardized using Plants of the World Online and Flora of Hawaiian Islands database (Wagner et al. 2005; POWO 2025). Species were classified based on their plant form. Reflecting the dominance of forests across the Hawaiian Islands (Barton et al. 2021), most species represented in the synthesis are woody (Table S2), sampled primarily in forests (98% of data points). In addition, the species pool includes three native fern species, and a greater diversity of growth forms for invasives: 2 fern, 1 climbing, 2 geophyte, 3 graminoid, and 1 herbaceous species (Table S2). A phylogenetic tree of the 42 plant species (Figure S1) was constructed based on Megatree using *get_tree* function in *rtree* package (Li 2023). To investigate non‐independence of litter traits due to shared evolutionary history, we tested for a phylogenetic signal using Blomberg's *K* (Blomberg and Garland 2002) and Pagel's *λ* (Pagel 1999) indices. Values of Blomberg's *K* and Pagel's *λ* equal to zero indicate weak phylogenetic signals, while values ≥ 1 indicate high phylogenetic signals. Additionally, we tested for *K* and *λ* statistical significance based on 1000 randomizations across the tips of the phylogenetic tree using *phylosig* function of *phytools* package in R. These analyses indicated weak phylogenetic signals for most traits (Table S4); therefore, we did not include phylogeny in the final models.

### 2.3 Climate Data

The locations of sampling sites were extracted from the original publications, and the range of sites captures the considerable environmental heterogeneity of the Hawaiian Islands, spanning from sites with very low (< 300 mm) to very high (> 6000 mm) mean annual rainfall, and from low (< 20 m) to high (> 2500 m) elevation (Figure 1). To investigate links between litter trait data and climate, we analyzed nine mean annual climate variables: air temperature (°C), surface temperature (°C), rainfall (mm), relative humidity (%), cloud cover (ratio), soil evaporation (mm), soil moisture (ratio), transpiration (mm), and soil radiation (W m^−2^). Site level climate data were extracted using QGIS software from the Hawaiʻi Climate Data Portal, which provides spatially interpolated climate data based on a 30‐year period 1987–2007 (Longman et al. 2024). Each climate variable was extracted at a 250 m resolution, which corresponds well to the scale at which litter trait data were collected.

### 2.4 Data Analysis

To investigate litter trait collinearity as a test for trait syndromes, we performed PCA on the nine litter functional traits. To avoid artificial data imputation for missing species trait data, we adopted the PCA approach suggested by Podani et al. (2021), in which correlations between each pair of traits were calculated by applying *pairwise.complete.obs* in the *cor* function in the R *stats* package. Next, the correlation matrix was used to compute the eigenvector matrix for traits PCA analysis (Podani et al. 2021). Redundancy analysis (RDA) was used to test whether plant origin (native vs. invasive) explains trait variation across multivariate trait space.

For additional insights, univariate analyses were conducted using one‐way analysis of variance (ANOVA) to test for native‐invasive differences for each of the litter traits. An ANOVA test was carried out using the *aov* function in R. Significant differences of litter traits between native vs. invasive were identified at *α* < 0.05 level. Because of its extensive representation in the dataset (49% of all data points, Table S2), a nonparametric Kernel density distribution was estimated to test the shift in litter traits values of endemic species ʻōhiʻa lehua ( *Metrosideros polymorpha* ) compared to invasive and other native species. The Kernel density was estimated using the *kde.test* function in the *ks* package in R.

To address data multicollinearity for climate, we conducted multivariate and correlation analyses. We assessed correlations between variables using Pearson's correlation and applied variance inflation factor (VIF) analysis using *vif* function in *usdm* package in R (R Core Team 2024). Variables with VIF scores greater than 10 were considered to indicate significant multicollinearity (Miles 2014). The VIF results showed that more than 50% of the climate variables used in our study were strongly multicollinear (Figure S2). Therefore, principal component analysis (PCA) was performed on climate variables and elevation, and PCA axis scores were used in analyses to represent climate.

To investigate whether native and invasive litter trait differences are stable across climate, we first tested for interactions between plant origin and climate when accounting for within‐species variation:

$$\begin{array}{c} \text{Model}\;1:\text{Trait}\sim \text{Plant origin}+\text{Plant origin}:\text{PC1}\\ \;+\text{Plant origin}:\text{PC2}+\left(1|\text{Study}\right)+\left(1|\text{Plant species}\right). \end{array}$$

where Trait is an individual litter trait (e.g., litter N, litter C), Plant origin is native or invasive, and PC1 and PC2 are the climate PCA axes scores. We included data source (Study ID) and Plant species as random factors. We then tested if the effects of plant origin and its interaction with climate on litter traits will be shifted when controlling for the main effects of climatic factors:

$$\begin{array}{c} \text{Model}\;2:\text{Trait}\sim \text{PC1}+\text{PC2}+\text{Plant origin}+\text{Plant origin}:\text{PC1}\\ \;+\text{Plant origin}:\text{PC2}+\left(1|\text{Study}\right)+\left(1|\text{Plant species}\right). \end{array}$$

However, we were not able to apply Model 1 and Model 2 for Ca.mass, Mg.mass and K.mass due to insufficient replication to address the full hierarchical models. Island was not included as a factor in our mixed models due to unbalanced sample sizes of native vs. invasive species across the islands. Notably, over 60% of the litter traits data used in this study were collected from the youngest island (Hawai‘i Island), with only 2 invasive species sampled on Moloka‘i, and no invasive species sampled on Maui or Kaua‘i (Figure 1). To investigate whether this unbalanced sample could drive trait comparison results, we conducted additional tests using only litter trait data sampled on Hawai‘i Island (Figures S3 and S4). These restricted results were qualitatively similar to the archipelago‐scale comparisons, and so we retain the full dataset for the analyses. Examination of trait‐climate relationships for different plant growth forms was inspected but not reported due to unbalanced replication (Figure S6).

To summarize the sources of variability in litter traits, we conducted a decomposition of variance analysis by fitting a linear mixed‐effects model across a nested classification hierarchy (Trait ~1 + (1|Plant origin/Plant form/Plant species)), and a variance component analysis was conducted using the *varcomp* function in the *ape* package in R. To avoid covariation among traits, the variance partitioning was performed trait‐by‐trait, while unexplained variance was used to account for within‐species variation.

## 3 Results

### 3.1 Litter Trait Variability Between Native and Invasive Plants

**FIGURE 2:**
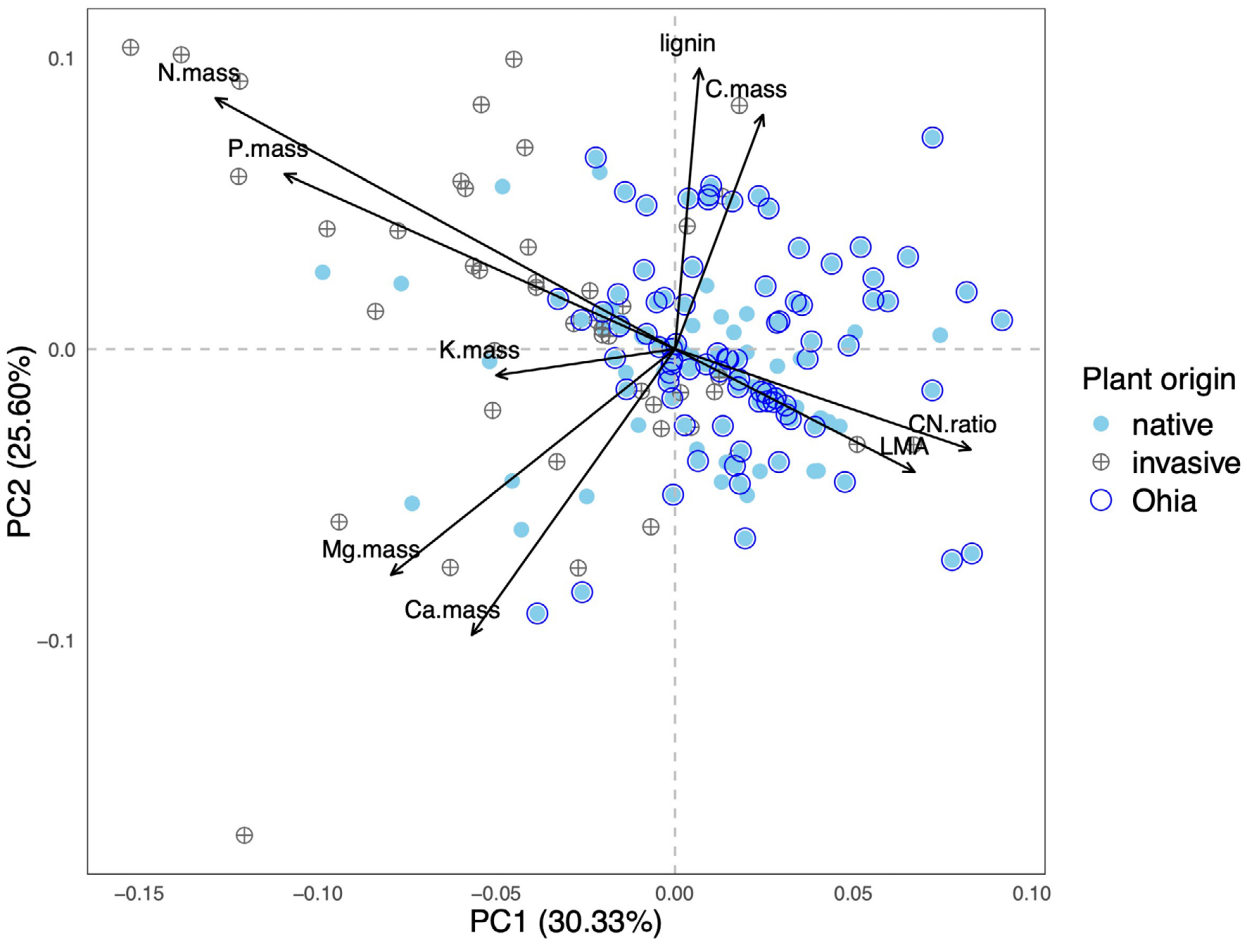
Multivariate litter trait space of native and invasive Hawaiian plant species characterized by nine key litter quality traits. The well‐sampled native plant species, ʻōhiʻa lehua ( *Metrosideros polymorpha* ), is marked to distinguish it from other native species. Each point is a species mean litter trait value from a sampled site.

**FIGURE 3:**
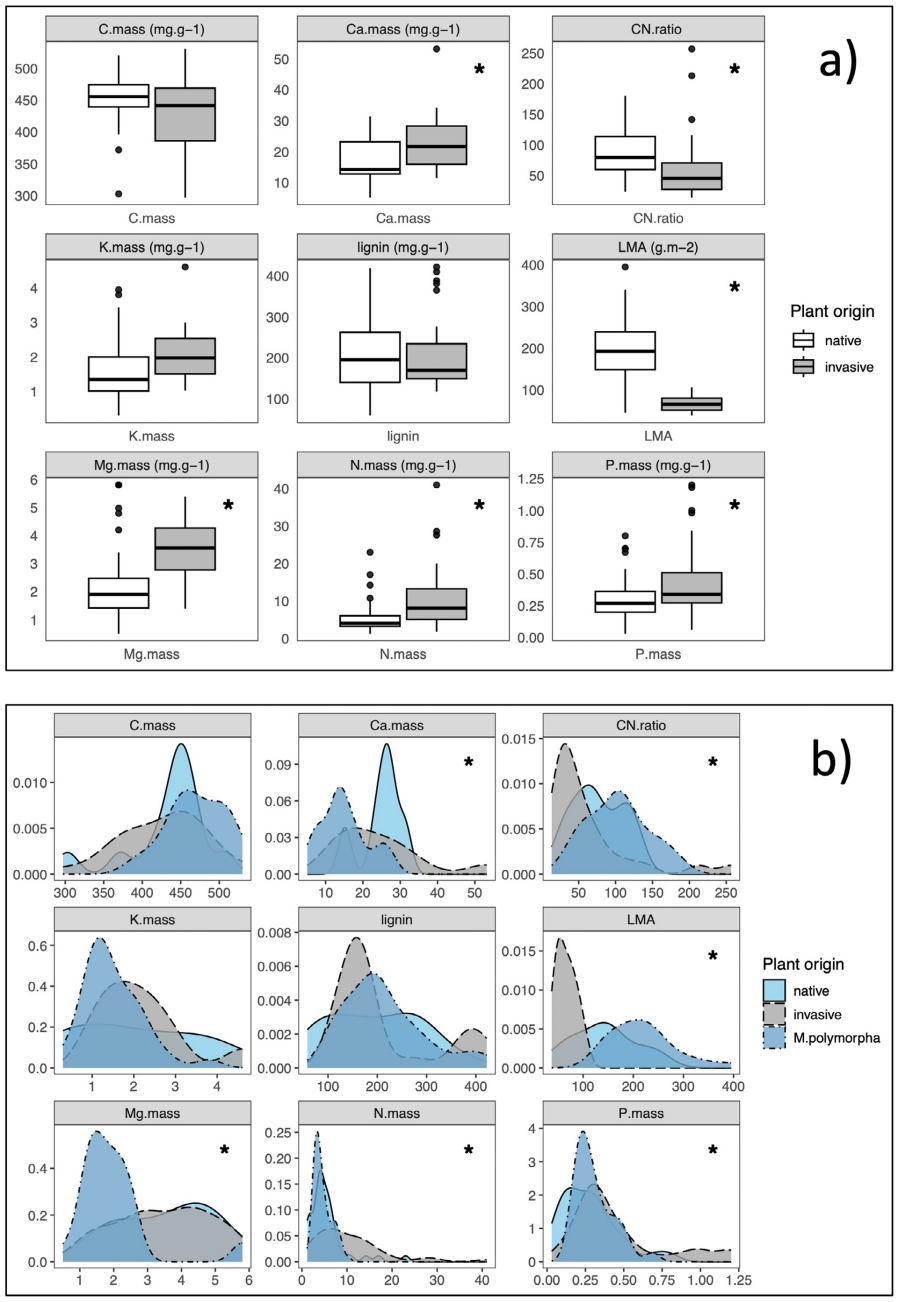
Results of one‐way ANOVA and Kernel density tests. (a) Summary of the raw litter traits data used in this study, *Significant ANOVA test results comparing the mean litter trait values between native and invasive species at *α* < 0.05. (b) Kernel density distributions of litter trait values comparing the endemic plant species ʻōhiʻa lehua ( *M. polymorpha* ), other native and invasive species, *the three distributions are statistically significant at *α* < 0.05.

Litter traits vary extensively, in both native and invasive species (Table S5). The PCA revealed trait collinearity along two axes, which accounts for a total of 55.93% of the total trait variance (Figure 2, Table S6). PC1 accounts for 30.33% of the total variance and is positively associated with LMA and C:N ratio and negatively associated with N.mass, P.mass, and Mg.mass. PC1 thus represents a continuum from high‐quality litter to low‐quality, tough leaf litter, corresponding to a shift from acquisitive to conservative plant resource‐use strategies and fast to slow litter decomposability. PC2 accounts for 25.60% of the total variance and is positively associated with lignin and C.mass and negatively associated with Ca.mass, Mg.mass, and LMA, representing an orthogonal litter quality dimension that spans from high to low quality based on distinct litter nutrient traits than PC1 (Figure 2, Table S6). The RDA analysis revealed that plant origin (native vs. invasive) is statistically significant (*p* = 0.001, *R* ^2^ = 0.074), but explains a relatively small percentage of total variance (variance RDA1 = 7.4%, *p* = 0.001). The univariate ANOVA analyses corroborate the multivariate analysis, revealing significant differences between native and invasive species for several litter traits (Figure 3).

Consistent with the prediction that invasive plants have faster, more acquisitive leaf functional strategies, invasive plant litter is significantly higher in nutrient content: P.mass (*F*_1,119_ = 13.6, *p* < 0.001), N.mass (*F*_1,151_ = 39.65, *p* < 0.001), Mg.mass (*F*_1,46_ = 7.27, *p* = 0.010), Ca.mass (*F*_1,46_ = 7.07, *p* = 0.01), and marginally lower in C:N (*F*_1,63_ = 4.05, *p* = 0.049). Invasive plant litter is also significantly less sclerophyllous than native plant litter (lower LMA, *F*_1,49_ = 40.89, *p* < 0.001) (Figure 3). There is some evidence that these native vs. invasive litter trait differences may be driven by litter data of the dominant endemic tree *M. polymorpha* , which deviates from other native species, particularly for leaf magnesium content (Figure 3b), and was also sampled much more extensively than other native species. The Kernel density analyses revealed that *M. polymorpha* has significantly lower N.mass but higher lignin and LMA compared to other native and invasive plant species (Figure 3b).

### 3.2 Climatic Gradients

The first two PC axes account for a total of 74.61% of the variance of the climatic variables (Figure S5). Climate PC axis 1 (PC1) accounts for 55.33% of the total variance and is positively associated with cloud cover, temperature, rainfall, soil moisture, humidity, and transpiration, and negatively associated with soil evaporation, solar radiation, and elevation (Table S7). PC1 thus represents a gradient from high elevation sites with cool and dry climates to low elevation sites with warm and wet climates (Figure S5). Climate PC axis 2 (PC2) explains 19.28% of the variance and is negatively associated with temperature but positively associated with rainfall, soil moisture, and humidity, representing an environmental continuum from warm, dry conditions to cool, wet conditions (Figure S5, Table S7). PC1 and PC2 scores were used in subsequent analyses to represent the site climate conditions.

### 3.3 Litter Trait and Climate

**FIGURE 4:**
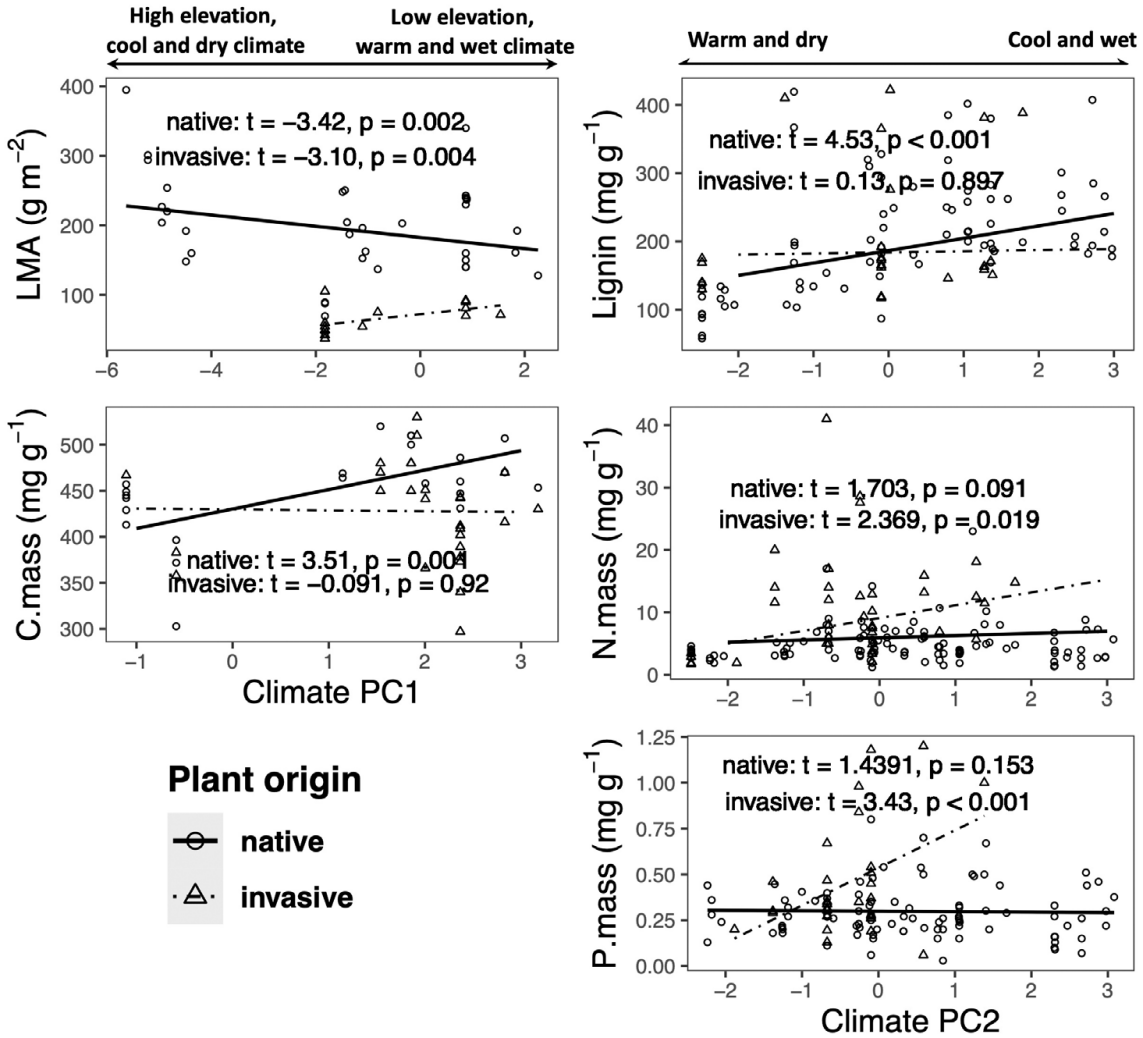
Relationships between litter traits and climatic factors, showing those traits with significant interactions between origin and climate.

As expected, there is evidence for significant relationships between litter traits and climate, but these patterns are not consistent for native and invasive species (Figure 4, Table S8). Litter LMA tends to decrease from cold, dry, high‐elevation sites to warm, wet, low‐elevation sites for native species, while the opposite pattern occurs for invasive species, resulting in weaker native‐invasive LMA differences at low elevation sites (Figure 4). C.mass significantly increases along the PC1 axis for native plants, but does not vary in invasive species, leading to a shift from trait convergence for native‐invasive litter at cold, dry, high‐elevation sites to an apparent difference in native‐invasive litter at warm, wet low‐elevation sites (Figure 4). Species origin interactions with climate PC2 were detected for N.mass and P.mass (Table S8), with higher nutrient content for invasive than native litter at cool, wet sites compared to trait convergence in warm, dry sites (Figure 4). Litter lignin content shifted across climate PC2 for native but not invasive species, leading to a reversal from lower lignin in invasive litter in cool, wet sites to lower lignin in native litter in warm, dry sites (Figure 4). Model 2 showed that, when controlling for the direct effects of climate (PC1 and PC2), plant origin was not a significant predictor of trait variation for several litter traits (lignin, LMA, P.mass and N.mass), suggesting that environmental factors such as climate play a more important role than species origin in shaping trait variation at the archipelago scale (Table S8).

### 3.4 Sources of Litter Trait Variability

**FIGURE 5:**
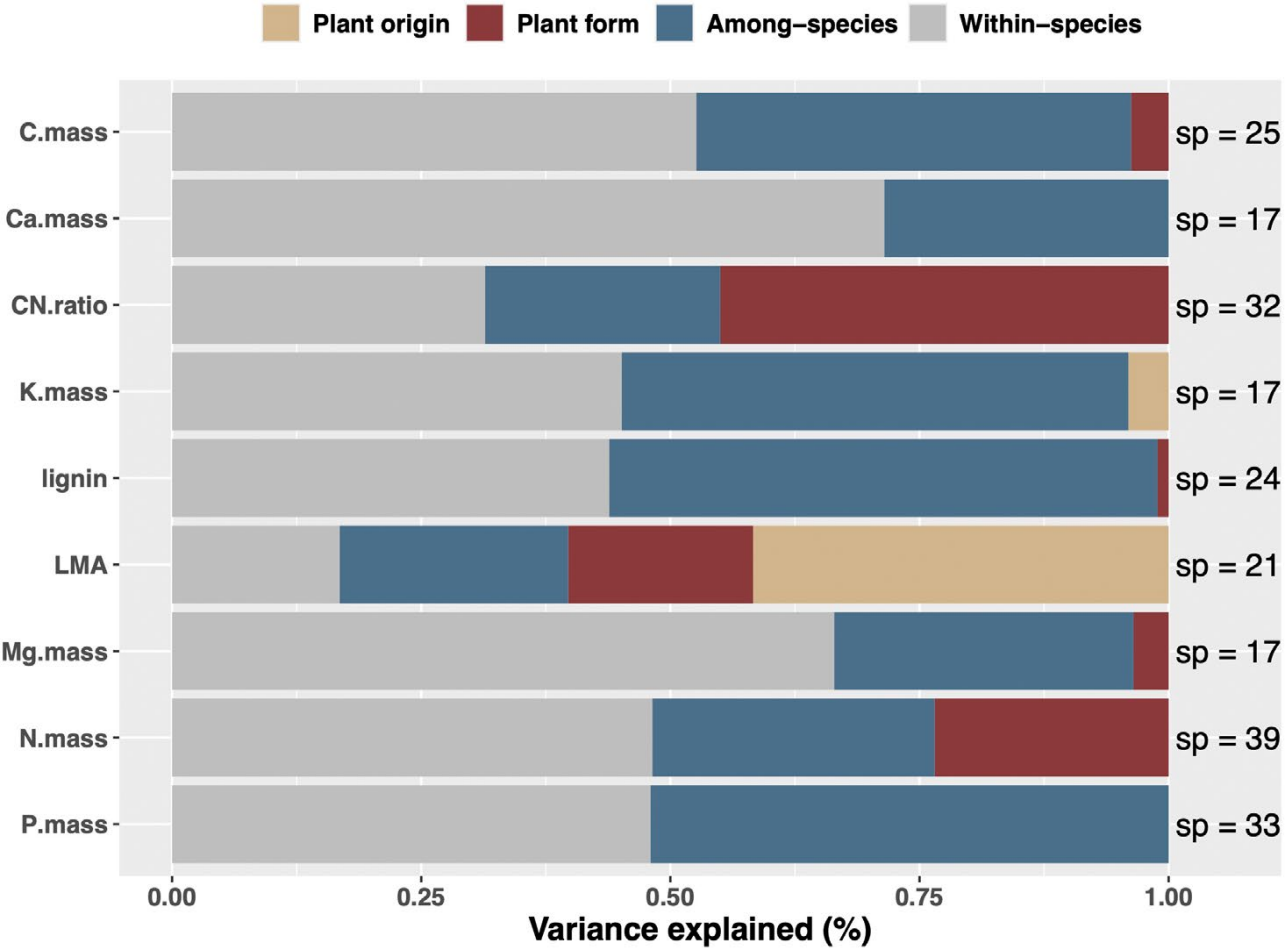
Major sources of litter trait variation for nine litter quality traits across the Hawaiian archipelago. Proportion of total variance explained by plant origin (native vs. invasive), plant form (woody, climbing, fern, herb, geophyte, and graminoid), species, and within species.

The variance decomposition analyses reveal different patterns across litter traits (Figure 5, Table S9). On average, intraspecific (47.10%) exceeds that of interspecific (37.20%) variation, although this varies across traits (Figure 5). Plant form accounts for a minor extent of trait variation at 10.60% on average, although N.mass and C:N ratio stood out as exceptions with plant form accounting for more than 50% of trait variation (Figure 5). Species origin generally accounts for only 5.08% of total trait variation, although for LMA, it is the largest source of variation (Figure 5, Table S9).

## 4 Discussion

The Hawaiian Islands have long served as a model system and living laboratory for the study of ecosystem and nutrient dynamics (P. M. Vitousek 1995, 2004). As invasive species continue to pose a serious threat, understanding the mechanisms by which invasives displace natives and disrupt ecosystem function is urgently needed. Because litter functional traits serve as both effect and response traits (Lavorel and Garnier 2002), they have the potential to be particularly informative. Litter's ability to serve in both roles complicates our understanding of nutrient cycling, and it should be noted that key soil characteristics such as soil fertility, soil texture, and soil biota, as well as their interactions with climate, were not assessed in this study. For example, soil nutrient availability has been shown to influence leaf litter quality in Hawaiian ecosystems (Hobbie and Vitousek 2000), and both low and high levels of soil nutrients can influence invasion patterns (Ostertag and Verville 2002; Funk and Vitousek 2007). Furthermore, soil biota vary greatly among tropical forests, with major differences in their relative importance (likely influenced by climate) on litter decomposition in tropical lowland vs. montane forests (Ostertag et al. 2022). These types of soil factors could not be examined in this synthesis because the studies from which the litter trait data were extracted failed to consistently report them.

Nevertheless, leveraging this well‐studied oceanic archipelago in which litter quality has been well characterized due to its role in nutrient cycling, we reveal extensive variability across broad climate gradients. As predicted, litter traits covary into syndromes that range from high (e.g., low LMA, high nutrient content) to low quality litter (high LMA, low nutrient content). This axis of litter quality corresponds to the well described leaf economics spectrum of live foliage, corroborating studies conducted elsewhere showing that litter traits are an echo of living leaf traits (Freschet et al. 2012). Whether species' positions along the spectrum are stable as leaves senesce depends on nutrient resorption rates, which were rarely reported in the studies synthesized here (Dudley et al. 2014). Nonetheless, litter trait covariance allows for a robust test for native‐invasive differences that may help explain the observed expansion of invasive plants in Hawaiʻi's native forests (Barton et al. 2021).

While litter trait data were compiled across broad climate gradients, this dataset nonetheless cannot represent the full scope of environments across the Hawaiian archipelago. Notably, litter trait sampling has been focused on the youngest island, Hawaiʻi, where there continues to be active volcanic activity, motivated by the goal of characterizing soil development and nutrient cycling during primary succession (Sherrod et al. 2021). Some native species litter sampling has been conducted on the older islands of Kauaʻi and Maui, but there is almost no invasive plant sampling on the older islands, and no litter quality data from Oʻahu for any species (Figure 1). Analyses restricted to Hawaiʻi Island produced qualitatively similar results to those reported here for the full dataset (Appendix S1), with the exception of lignin which had minor differences with respect to climate variability (Figure S4), suggesting that the unbalanced representation of native vs. invasive sampling across the islands does not drive the main results. Nonetheless, this synthesis potentially depicts only a partial characterization of the full scope of litter trait variability, with intermediate age substrates under‐represented. Along the 4.1‐million‐year‐old chronosequence of the main Hawaiian Islands, we might predict the greatest convergence of native and invasive species on high‐quality litter on intermediate age substrates as nutrient limitation does not constrain performance as it does on the youngest and oldest substrates (Crews et al. 1995; Vitousek 1995). However, nutrient limitation is not only related to substrate age, but also to soil chemistry and texture, climatic conditions, and disturbance (Vitousek 2004; Vitousek et al. 1998). Additional sampling of litter of both native and invasive species growing on a range of substrate ages, but most especially on the intermediate and older islands, is needed to address this knowledge gap.

### 4.1 Extensive Litter Trait Variability Obscures Native‐Invasive Differences

We predicted that invasive plant litter would be high‐quality, with high mineral content and low toughness, associated with fast resource use and life history strategies, while native plant litter would be low quality as a consequence of a more conservative resource‐use strategy (Lee et al. 2017; Liao et al. 2008). We detected some support for these predictions, particularly for LMA, which was significantly higher in native than in invasive litter. Tough litter with high LMA is generally associated with slow decomposition rates (Cornwell et al. 2008), and so this result indicates that invaded sites in Hawaiʻi are likely to experience faster decomposition rates as a consequence of invasive plant litter production. However, LMA alone does not drive decomposition rates, which are also highly dependent on litter chemistry via mineral content and lignin (Cornwell et al. 2008), as well as environmental context including climate and soil fauna and microbiota (Bourget et al. 2023; Zhang et al. 2008).

Litter chemistry was highly variable within native and invasive species, suggesting a range of acquisitive to conservative resource‐use strategies within both groups. Nonetheless, the univariate analyses revealed that invasive litter tends to be of higher quality, with significantly higher magnesium, nitrogen, and phosphorus content, and lower C:N ratios than native plant litter. In contrast, carbon, potassium, and lignin content were similar in native and invasive species. There is some evidence that the significant differences detected between native and invasive species are at least partly driven by a single endemic tree species, *M. polymorpha* , which accounts for 68% of the native plant data points (336/495 litter trait means), and is well known to have a conservative resource‐use strategy and a slow life history (Hart 2010; Cordell et al. 1998). A research focus on *M. polymorpha* reflects its importance in Hawaiʻi's forests, as the most widespread and abundant native tree, particularly on Hawaiʻi Island, where the vast majority of litter trait sampling has been conducted (92% of the 631 total data points for which sampling location was reported). However, it is not representative of the entire flora, and with trait data from only 17 native species included in this synthesis, it is premature to attempt to characterize general patterns in litter trait quality for the approximately 1400 native Hawaiian plant species (Wagner et al. 2005).

The differences between native and invasive plant litter traits detected with univariate analyses become much more subtle when examining multivariate trait collinearity. Invasive and native species occur throughout the multivariate trait space parameterized by the PCA, particularly when considering native species other than *M. polymorpha* . While the redundancy analysis corroborates the univariate analyses, reporting a statistically significant difference between native and invasive species traits, species origin accounts for less than 10% of trait variance. Thus, while there is some evidence to support the prediction that invasive plants have higher quality litter, the magnitude of this difference is small compared to other sources of trait variability, such as species identity, growth form, and climate. Interestingly, these results are consistent with the growing consensus that Hawaiʻi's native plants have evolved a great diversity of resource‐use strategies, with minimal differences in live leaf traits compared to non‐native plant species established in Hawaiʻi (Westerband et al. 2021; Henn et al. 2019) or compared to native species sampled in continental habitats (Westerband et al. 2024). Similar patterns have been reported for other archipelagoes in which native and invasive plants have diverse and overlapping resource‐use functional traits (Kühn et al. 2021). Such extensive diversity in resource‐use functional traits should not be unexpected, even for a highly endemic flora like that of Hawaiʻi that diversified from a mere 259 colonizing lineages (Price and Wagner 2018), because adaptive radiations and intraspecific variation resulting from local adaptation in widespread species across steep environmental gradients are commonly associated with high phenotypic diversity (Kuppler et al. 2020; Schenk 2021).

While origin generally explains relatively little variability in litter traits, other factors emerged as more important drivers of trait variability, including plant growth form and climate. In particular, litter carbon, nitrogen, and their ratio vary predominantly with respect to plant growth form, mainly as a result of the very high C:N ratios reported for invasive grass litter (Mack and D'Antonio 2003). Native tree ferns also tend to have relatively high C:N ratios (Funk 2005) and slow rates of decomposition (Amatangelo and Vitousek 2009). The variability in litter chemistry we detected across growth forms corresponds to global patterns of slow to fast decomposition rates found for ferns, monocots, and dicots (Cornwell et al. 2008). Importantly, growth form interacts with climate in Hawaiʻi in non‐random ways. For example, grasses are the predominant invader in dryland and mesic sites (D'Antonio et al. 2011), which also corresponds to climatic conditions good for cattle production (Faccenda 2025), while high elevation alpine areas tend to be invaded by herbaceous species of temperate origin (Daehler 2005). The consequence of this non‐random distribution of invaders means that growth form variability in litter quality and decomposition are associated with a turn‐over in physiognomy and architecture in Hawaiian ecosystems. For example, native ferns are an important component of Hawaiian forest understories (Ranker 2016), and they are being displaced by invasive grasses with lower quality litter that now dominate the forest understories in mesic and dry habitats (D'Antonio et al. 2017). At the same time, invasive nitrogen‐fixing trees and shrubs (e.g., *Prosopis pallida* , *Falcataria falcata*, *Morella faya* ) produce very high quality litter, facilitating their establishment in nitrogen‐limited, young volcanic soils in both wet forests and former dry forest habitats (Vitousek et al. 1987). Such shifts in plant functional traits via turn‐over in growth forms have been documented in other parts of the world, illustrating the importance of this invasion dynamic (Funk et al. 2016). Characterizing plant litter quality for insights into plant invasion in Hawaiʻi thus requires consideration of plant growth form and the complex dynamics of community turn‐over in physiognomy as well as species identity.

### 4.2 Climate Heterogeneity Underlies Litter Trait Variability

Climate drives litter decomposition directly via effects on the decomposer community composition and activity as well as indirectly via effects on litter quality via local adaptation of plant resource economic strategies that include litter traits. These processes are linked so that climate history can influence litter production and decomposition rates in complex ways, for example, by priming decomposer activity in response to deviations from historical mean conditions of water availability and temperature (Strickland et al. 2015). Moreover, litter quality that evolved as part of plant resource economic strategies may then interact with climate variability during decomposition, leading to dynamic relationships between litter quality and decomposition over time (Canessa et al. 2021). Thus, to characterize how climate controls decomposition, it would be important to include both historical and ambient metrics of climate variables. In this synthesis, we lack the ambient climate data, and so instead focus on potential evolutionary links between litter quality traits and historical climate records.

Litter quality shifted significantly with climate, likely in response to interspecific variation via species turn‐over across the landscape, as well as intraspecific variation due to phenotypic plasticity and local adaptation. Moreover, climate‐trait links varied in distinct ways for native and invasive species, providing key insights into native‐invasive functional trait strategies in Hawaiʻi. Climate is well documented to be a major driver of the evolution of plant functional traits for both live foliage as well as litter quality (Yuan et al. 2024; Wright et al. 2005). That native‐invasive trait differences can vary across climate gradients has previously been documented in Hawaiʻi in a study of leaf economics spectrum traits of live foliage (Westerband et al. 2021). Comparing traits sampled from 60 native and 31 invasive woody plant species, it emerged that invasives tend to have more acquisitive strategies in wet, cool sites while natives tend to have more acquisitive strategies at arid, hot sites (Westerband et al. 2021). For the litter traits synthesized here, the interactions detected between species origin and climate largely reflected variable magnitudes of change in litter quality across climate. For example, invasive species have higher quality litter (on the basis of nitrogen and phosphorus content) at cool, wet sites than warm, dry sites, while native litter quality tended to vary little across climate. However, for a single litter trait, lignin, a reversal in functional strategy was observed such that invasives tend to have higher quality litter (i.e., lower lignin) than natives in cool, wet sites while natives had higher quality litter than invasives in warm, dry sites, a similar pattern to that of the live foliage traits examined in Westerband et al. (2021).

That invasive species are more acquisitive with higher quality litter than native species in only a small subset of climates (i.e., cool, wet) does not support the prediction that displacement via resource‐use strategies with feedback from litter decomposition underlies invasive plant spread across the Hawaiian Archipelago. Instead, it seems likely that this resource‐use dynamic underlies the establishment of only some invasive species at some sites. Other factors, such as habitat loss and fragmentation, lost mutualists, climate change, human commensalism, and novel natural enemies are likely to be more important drivers of native plant declines in Hawaiʻi at many sites. Furthermore, interactive effects of these global change threats in combination with invasive species are likely to be important and deserving of future research for a better understanding of native plant declines.

## 5 Conclusion

Litter quality traits are under‐represented in trait‐based ecology, despite their clear importance for nutrient cycling and plant productivity. Leveraging a dataset representing 30 years of litter research in Hawaiʻi, we demonstrate here that litter quality traits covary into syndromes very similar to the leaf economics spectrum of live foliage traits, indicating the importance of litter quality as an extension of the living phenotype. We reveal significant, but subtle, differences in litter traits between native and invasive species, offering some support for the prediction that invasives displace natives via fast resource use and high‐quality litter that decomposes quickly. However, these differences are contingent on climate, corroborating studies on live plant traits showing a broad diversity of functional strategies for both native and invasive plants in Hawaiʻi. Furthermore, the dataset, though representative of an impressive body of research, remains incomplete, with focus dominated by a single species, *M. polymorpha* , sampled predominantly on a single island. Future research is needed to expand our understanding of litter quality within the native species and across the steep climate gradients and variable soil substrates of the Hawaiian Archipelago.

## Author Contributions

**Manichanh Satdichanh:** conceptualization (equal), data curation (lead), formal analysis (lead), methodology (equal), writing – original draft (equal), writing – review and editing (equal). **Rebecca Ostertag:** conceptualization (supporting), funding acquisition (equal), writing – review and editing (supporting). **William Harrigan:** data curation (supporting), writing – review and editing (supporting). **Mahdi Belcaid:** formal analysis (supporting), funding acquisition (supporting), writing – review and editing (supporting). **Kasey E. Barton:** conceptualization (lead), data curation (equal), funding acquisition (equal), project administration (equal), supervision (lead), writing – original draft (equal), writing – review and editing (equal).

## Funding

This work was supported by the National Science Foundation (OIA‐2149133).

## Conflicts of Interest

The authors declare no conflicts of interest.

## Supporting information


**Appendix S1:** ece373030‐sup‐0001‐AppendixS1.docx.

## Data Availability

The raw data have been submitted for review, and will be archived in Dryad upon publication.
