# Supplementary material for: Plant Litter Trait Variation Between Native and Invasive Species Across Steep Climate Gradients in the Hawaiian Islands
Source: Ecol Evol. 2026 Feb 9;16(2):e73030. doi: 10.1002/ece3.73030 (PMC12885483; doi:10.1002/ece3.73030)
Supplement: Supplementary file 1 — Appendix S1: ece373030‐sup‐0001‐AppendixS1.docx. [file ECE3-16-e73030-s001.docx]

**Plant litter trait variation between native and invasive species across steep climate gradients in the Hawaiian Islands**

Manichanh Satdichanh^1^, Rebecca Ostertag^2^, William Harrigan^3^, Mahdi Belcaid^3,4^, ﻿ and Kasey E. Barton^1,*^

^1^ School of Life Sciences, University of Hawaiʻi at Mānoa, Honolulu, Hawaiʻi 96822, USA

^2^ Department of Biology, University of Hawaiʻi at Hilo, Hawaiʻi 96720, USA

^3^ Hawaiʻi Institute of Marine Biology, University of Hawaiʻi at Mānoa﻿, Honolulu, Hawaiʻi 96744, USA

^4^ Department of Computer Science, University of Hawaiʻi at Mānoa, Honolulu, HI, 96822, USA

* Correspondence: [kbarton@hawaii.edu](mailto:kbarton@hawaii.edu). https://orcid.org/0000-0002-4549-4150

**Appendix**

- Systematic Review Methods
- Data Sources – List of publications with data used in analyses
- Table A1 – Overview of litter traits included in synthesis, including typical unit of measurement and ecological importance.
- Table A2 – Summary of species included in analyses. Plant growth form is specified for woody plants for trees (T) and shrubs (S), and for tree ferns (TF). The total number of replicates across all traits is reported for each species.
- Table A3 – Total sample sizes for nine litter traits used in this study. Each replicate is a trait mean sampled from a species at a given site. There can be multiple replicates per species if they were sampled at multiple sites or by multiple studies.
- Table A4 – Results from the tests for phylogenetic signal (Bloomberg’s K and Pagel’s λ) of litter traits, including both native and invasive species. Traits with significant phylogenetic signals are highlighted in bold font.
- Table A5 – Litter trait data range for native and invasive plant species across all sites.
- Table A6 – Results from the multivariate analyses of litter traits. Loading scores of the 9 litter traits from the principal component analysis (PCA) are reported.
- Table A7 – Results from the multivariate analyses of climate across the study sites where litter trait data were sampled. Loading scores of the 9 climate variables and elevation from the principal component analysis (PCA) are reported.
- Table A8 – Summary results of the Mixed Random Effect models. Only traits with significant results are reported. Model 1 is to test for the effect of plant origin and its interactions with climate on litter traits when accounting for within-species variation, and Model 2 is to test if these effects shifted when controlling for the full effects of climate factors when accounting for within-species variation.
- Table A9 – Results of decomposition of variance analysis. Percentage of variance accounted by plant form, plant origin, among and within species for each litter trait.
- Figure A1 – Phylogenetic tree of 42 plant species used in this study. Black = native species, red = invasive species.
- Figure A2 – Pearson’s correlation and variance inflation factor scores of the climatic variables. The dashed red line represents a VIF threshold of ≥10, indicating significant multicollinearity among the climate variables.
- Figure A3 – Multivariate trait space of native and invasive plant species, using only data sampled on Hawaiʻi Island, characterized by nine key litter quality traits. The dominant native tree species ʻōhiʻa lehua (*M. polymorpha*) is also indicated due to its dominance in the data set.
- Figure A4 – Results of one-way ANOVA and Kernel density tests for trait data sampled only on Hawai‘i Island. a) summary of the raw litter traits data used in this study, * significant ANOVA test results comparing the mean litter trait values between native and invasive species at α < 0.05. b) Kernel density distributions of litter trait values comparing the endemic plant species ʻōhiʻa lehua (*M. polymorpha*), other native and invasive species, * the three distributions are statically significant at α < 0.05.
- Figure A5 – Principal component analysis (PCA) plot of climatic variables used in this study.
- Figure A6 – Relationship between litter traits and climatic factors, showing those traits with significant interactions between origin and climate. Growth form is designated for each species.

**Systematic Review Methods**

Studies were identified through searches conducted on Web of Science Core Collection (1980-2023) and Google Scholar in January 2023, using concatenated strings of terms: (Trait OR morpholog* OR physiolog* OR anatom* OR hydraul* OR pollinat* OR defen?e OR decomposition OR growth OR reproduc*) AND Hawai* AND (leaf OR root OR wood OR stem OR flower OR seed OR litter OR architectur*) AND (plant OR vegetat* OR grass OR herb OR tree OR fern). In addition, forward and reverse searches were conducted to identify relevant studies cited in or citing those identified through the original searches. These searches yielded a total of 2,007 potentially relevant publications, which were assessed and reduced to 35 that met all inclusion criteria.

**Data Sources – Publications with data extracted for synthesis**

Allison, S. D., & Vitousek, P. M. (2004). Extracellular Enzyme Activities and Carbon Chemistry as Drivers of Tropical Plant Litter Decomposition. *Biotropica*, *36*(3), 285–296. https://doi.org/10.1111/j.1744-7429.2004.tb00321.x

Amatangelo, K. L., & Vitousek, P. M. (2009). Contrasting Predictors of Fern versus Angiosperm Decomposition in a Common Garden. *Biotropica*, *41*(2), 154–161. https://doi.org/10.1111/j.1744-7429.2008.00470.x

Ares, A., & Fownes, J. H. (2001). Productivity, resource use, and competitive interactions of Fraxinus uhdei in Hawaii uplands. *Canadian Journal of Forest Research*, *31*(1), 132–142. https://doi.org/10.1139/cjfr-31-1-132

Asner, G. P., & Beatty, S. W. (1996). Effects of an African grass invasion on Hawaiian shrubland nitrogen biogeochemistry. *Plant and Soil*, *186*(2), 205–211. https://doi.org/10.1007/BF02415515

Austin, A. T. (2002). Differential effects of precipitation on production and decomposition along a rainfall gradient in Hawaii. *Ecology*, *83*(2), 328–338. https://doi.org/https://doi.org/10.1890/0012-9658(2002)083[0328:DEOPOP]2.0.CO;2

Austin, A. T., & Vitousek, P. M. (2000). Precipitation, decomposition and litter decomposability of *Metrosideros polymorpha* in native forests on Hawai’i. *Journal of Ecology*, *88*(1), 129–138. https://doi.org/10.1046/j.1365-2745.2000.00437.x

Bothwell, L. D., Selmants, P. C., Giardina, C. P., & Litton, C. M. (2014). Leaf litter decomposition rates increase with rising mean annual temperature in Hawaiian tropical montane wet forests. *PeerJ*, *2*, e685. https://doi.org/10.7717/peerj.685

Chau, M. M., Walker, L. R., & Mehltreter, K. (2013). An invasive tree fern alters soil and plant nutrient dynamics in Hawaii. *Biological Invasions*, *15*(2), 355–370. https://doi.org/10.1007/s10530-012-0291-0

Crews, T. E., Kitayama, K., Fownes, J. H., Riley, R. H., Herbert, D. A., Mueller-Dombois, D., & Vitousek, P. M. (1995). Changes in soil phosphorus fractions and ecosystem dynamics across a long chronosequence in Hawaii. *Ecology*, *76*(5), 1407–1424. https://doi.org/10.2307/1938144

Funk, J. L. (2005). *Hedychium gardnerianum* invasion into Hawaiian montane rainforest: Interactions among litter quality, decomposition rate, and soil nitrogen availability. *Biogeochemistry*, *76*(3), 441–451. https://doi.org/10.1007/s10533-005-7657-7

Funk, J. L., & Amatangelo, K. L. (2013). Physiological mechanisms drive differing foliar calcium content in ferns and angiosperms. *Oecologia*, *173*(1), 23–32. https://doi.org/10.1007/s00442-013-2591-1

Funk, J. L., & McDaniel, S. (2010). Altering Light Availability to Restore Invaded Forest: The Predictive Role of Plant Traits. *Restoration Ecology*, *18*(6), 865–872. https://doi.org/10.1111/j.1526-100X.2008.00515.x

Harrington, R. A., Fownes, J. H., & Vitousek, P. M. (2001). Production and resource use efficiencies in N- and P-limited tropical forests: A comparison of responses to long-term fertilization. *Ecosystems*, *4*(7), 646–657. https://doi.org/10.1007/s10021-001-0034-z

Hättenschwiler, S., Hagerman, A. E., & Vitousek, P. M. (2003). Polyphenols in litter from tropical montane forests across a wide range in soil fertility. *Biogeochemistry*, *64*(1), 129–148. https://doi.org/10.1023/A:1024966026225

Herbert, D. A., & Fownes, J. H. (1995). Phosphorus limitation of forest leaf area and net primary production on a highly weathered soil. *Biogeochemistry*, *29*(3), 223–235. https://www.jstor.org/stable/1468922

Herbert, D. A., Fownes, J. H., & Vitousek, P. M. (1999). Hurricane damage to a Hawaiian forest: Nutrient supply rate affects resistance and resilience. *Ecology*, *80*(3), 908–920. https://doi.org/10.1890/0012-9658(1999)080[0908:HDTAHF]2.0.CO;2

Hobbie, S. E. (2000). Interactions between litter lignin and soil nitrogen availability during leaf litter decomposition in a Hawaiian montane forest. *Ecosystems*, *3*(5), 484–494. https://doi.org/10.1007/s100210000042

Hughes, R. F., & Uowolo, A. (2006). Impacts of *Falcataria moluccana* invasion on decomposition in Hawaiian lowland wet forests: The importance of stand-level controls. *Ecosystems*, *9*(6), 977–991. https://doi.org/10.1007/s10021-005-0083-9

Ley, R. E., & D’Antonio, C. M. (1998). Exotic grass invasion alters potential rates of N fixation in Hawaiian woodlands. *Oecologia*, *113*(2), 179–187. https://doi.org/10.1007/s004420050366

Mack, M. C., & D’Antonio, C. M. (2003). The effects of exotic grasses on litter decomposition in a Hawaiian woodland: the importance of indirect effects. *Ecosystems*, *6*(8), 723–738. https://doi.org/10.1007/s10021-003-0119-y

MacKenzie, R. A., Wiegner, T. N., Kinslow, F., Cormier, N., & Strauch, A. M. (2013). Leaf-litter inputs from an invasive nitrogen-fixing tree influence organic-matter dynamics and nitrogen inputs in a Hawaiian river. *Freshwater Science*, *32*(3), 1036–1052. https://doi.org/10.1899/12-152.1

Mascaro, J., Hughes, R. F., & Schnitzer, S. A. (2012). Novel forests maintain ecosystem processes after the decline of native tree species. *Ecological Monographs*, *82*(2), 221–228. https://doi.org/10.1890/11-1014.1

Matson, P. (1990). Plant-soil interactions in primary succession at Hawaii Volcanoes National Park. *Oecologia*, *85*(2), 241–246. https://doi.org/10.1007/BF00319408

Ostertag, R., Cordell, S., Michaud, J., Cole, T. C., Schulten, J. R., Publico, K. M., & Enoka, J. H. (2009). Ecosystem and restoration consequences of invasive woody species removal in Hawaiian lowland wet forest. *Ecosystems*, *12*(3), 503–515. https://doi.org/10.1007/s10021-009-9239-3

Ostertag, R., & Hobbie, S. E. (1999). Early stages of root and leaf decomposition in Hawaiian forests: Effects of nutrient availability. *Oecologia*, *121*(4), 564–573. https://doi.org/10.1007/s004420050963

Pearson, H. L., & Vitousek, P. M. (2001). Stand dynamics, nitrogen accumulation, and symbiotic nitrogen fixation in regenerating stands of *Acacia koa*. *Ecological Applications*, *11*(5), 1381–1394. https://doi.org/10.1890/1051-0761(2001)011[1381:SDNAAS]2.0.CO;2

Pérez, F. L. (2019). Plant organic matter really matters: Pedological effects of kūpaoa (dubautia menziesii) shrubs in a volcanic alpine area, maui, Hawai’i. *Soil Systems*, *3*(2), 1–29. https://doi.org/10.3390/soilsystems3020031

Riley, R. H., & Vitousek, P. M. (1995). Nutrient dynamics and nitrogen trace gas flux during ecosystem development in montane rain forest. *Ecology*, *76*(1), 292–304. https://doi.org/10.2307/1940650

Roberts, M., Strauch, A. M., Wiegner, T., & MacKenzie, R. A. (2016). Leaf litter breakdown of native and exotic tree species in two Hawaiian streams that differ in flow. *Pacific Science*, *70*(2), 209–222. https://doi.org/10.2984/70.2.7

Rothstein, D. E., Vitousek, P. M., & Simmons, B. L. (2004). An exotic tree alters decomposition and nutrient cycling in a Hawaiian montane forest. *Ecosystems*, *7*(8), 805–814. https://doi.org/10.1007/s10021-004-0009-y

Russell, A. E., Raich, J. W., & Vitousek, P. M. (1998). The ecology of the climbing fern *Dicranopteris linearis* on windward Mauna Loa, Hawai’i. *Journal of Ecology*, *86*(5), 765–779.

Russell, A. E., & Vitousek, P. M. (1997). Decomposition and potential nitrogen fixation in *Dicranopteris linearis* litter on Mauna Loa, Hawai’i. *Journal of Tropical Ecology*, *13*(4), 579–594. https://doi.org/10.1017/S0266467400010737

Scowcroft, P. G. (1997). Mass and nutrient dynamics of decaying litter from *Passiflora mollissima* and selected native species in a Hawaiian montane rain forest. *Journal of Tropical Ecology*, *13*(3), 407–426. https://doi.org/10.1017/S0266467400010592

Scowcroft, P. G., & Jeffrey, J. (1999). Potential significance of frost, topographic relief, and *Acacia koa* stands to restoration of mesic Hawaiian forests on abandoned rangeland. *Forest Ecology and Management*, *114*(2–3), 447–458. https://doi.org/10.1016/S0378-1127(98)00374-0

Sin, H., Beard, K. H., & Pitt, W. C. (2008). An invasive frog, *Eleutherodactylus coqui*, increases new leaf production and leaf litter decomposition rates through nutrient cycling in Hawai’i. *Biological Invasions*, *10*(3), 335–345. https://doi.org/10.1007/s10530-007-9133-x

Stewart, C. E., Neff, J. C., Amatangelo, K. L., & Vitousek, P. M. (2011). Vegetation effects on soil organic matter chemistry of aggregate fractions in a Hawaiian forest. *Ecosystems*, *14*(3), 382–397. https://doi.org/10.1007/s10021-011-9417-y

Vitousek, P. M., Gerrish, G., Turner, D. R., Walker, L. R., & Mueller-Dombois, D. (1995). Litterfall and nutrient cycling in four Hawaiian montane rainforests. *Journal of Tropical Ecology*, *11*(2), 189–203. https://doi.org/10.1017/S0266467400008634

Vitousek, P. M., Walker, L. R., Whiteaker, L. D., & Matson, P. A. (1993). Nutrient limitations to plant growth during primary succession in Hawai’i Volcanoes National Park. *Biogeochemistry*, *23*(3), 197–215. https://doi.org/10.1007/BF00023752

Vitousek, P. M. (1998). Foliar and litter nutrients, nutrient resorption, and decomposition in Hawaiian *Metrosideros polymorpha*. *Ecosystems*, *1*(4), 401–407. https://doi.org/10.1007/s100219900033

Vitousek, P. M., & Hobbie, S. (2000). Heterotrophic nitrogen fixation in decomposing litter: Patterns and regulation. *Ecology*, *81*(9), 2366–2376. https://doi.org/10.1890/0012-9658(2000)081[2366:HNFIDL]2.0.CO;2

Vitousek, P. M., Turner, D. R., & Kitayama, K. (1995). Foliar nutrients during long‐term soil development in Hawaiian montane rain forest. *Ecology*, *76*(3), 712–720. https://doi.org/10.2307/1939338

Vitousek, P. M., Turner, D. R., Parton, W. J., & Sanford, R. L. (1994). Litter decomposition on the Mauna Loa environmental matrix, Hawaiʻi: patterns, mechanisms, and models. *Ecology*, *75*(2), 418–429. https://doi.org/10.2307/1939545

Vitousek, P. M., & Walker, L. R. (1989). Biological invasion by *Myrica faya* in Hawai’i: plant demography, nitrogen fixation, ecosystem effects. *Ecological Monographs*, *59*(3), 247–265. https://doi.org/10.2307/1942601

Wardle, D. A., Bardgett, R. D., Walker, L. R., & Bonner, K. I. (2009). Among- and within-species variation in plant litter decomposition in contrasting long-term chronosequences. *Functional Ecology*, *23*(2), 442–453. <https://doi.org/10.1111/j.1365-2435.2008.01513.x>

Wiegner, T. N., & Tubal, R. L. (2010). Comparison of dissolved organic carbon bioavailability from native and invasive vegetation along a Hawaiian river. *Pacific Science*, *64*(4), 545–555. https://doi.org/10.2984/64.4.545

**Table A1** Overview of litter traits included in synthesis, including typical unit of measurement and ecological importance.

| Litter trait | Unit | Ecological importance |
| --- | --- | --- |
| Lignin content | mg.g^-1^ | Key characteristics of plant litter in regulating decomposition and organic matter turnover rates that affecting nutrient cycling and carbon storage (Austin & Ballaré, 2010) |
| Leaf mass per area of litter (LMA) | g.m^-2^ | Higher LMA is associated with tougher leaves, lower nutrient content, and slower decomposition |
| Carbon and nitrogen ratio (C:N ratio) |  | Higher C:N ratio indicates low quality litter, lower nutrient content, and slower decomposition |
| Nitrogen concentration (N.mass) | mg.g^-1^ | High litter nitrogen and phosphorus content promotes faster decomposition, influences microbial community and nutrient cycling (Hobbie, 2015; Manzoni et al., 2008) |
| Phosphorus concentration (P.mass) | mg.g^-1^ |  |
| Carbon concentration (C.mass) | mg.g^-1^ | Primary source of soil organic matter, facilitating decomposition processes (Lyu et al., 2023) |
| Calcium concentration (Ca.mass) | mg.g^-1^ | Key physiological traits, influences litter decomposition processes, soil microbial community and soil pH(Zhou et al., 2021) |
| Magnesium concentration (Mg.mass) | mg.g^-1^ |  |
| Potassium concentration (K.mass) | mg.g^-1^ |  |

**References:**

Austin, A. T., & Ballaré, C. L. (2010). Dual role of lignin in plant litter decomposition in terrestrial ecosystems. *Proceedings of the National Academy of Sciences of the United States of America*, *107*(10), 4618–4622. https://doi.org/10.1073/pnas.0909396107

Hobbie, S. E. (2015). Plant species effects on nutrient cycling: revisiting litter feedbacks. *Trends in Ecology and Evolution*, *30*(6), 357–363. https://doi.org/10.1016/j.tree.2015.03.015

Lyu, M., Homyak, P. M., Xie, J., Peñuelas, J., Ryan, M. G., Xiong, X., Sardans, J., Lin, W., Wang, M., Chen, G., & Yang, Y. (2023). Litter quality controls tradeoffs in soil carbon decomposition and replenishment in a subtropical forest. *Journal of Ecology*, *111*(10), 2181–2193. https://doi.org/10.1111/1365-2745.14167

Manzoni, S., Jackson, R. B., Trofymow, J. A., & Porporato, A. (2008). The global stoichiometry of litter nitrogen mineralization. *Science*, *321*(5889), 684–686. https://doi.org/10.1126/science.1159792

Zhou, S., Yan, G., Hu, J., Liu, X., Zou, X., Tie, L., Yuan, R., Yang, Y., Xiao, L., Cui, X., Tu, L., Lai, J., Zhao, A., & Huang, C. (2021). The responses of leaf litter calcium, magnesium, and manganese dynamics to simulated nitrogen deposition and reduced precipitation vary with different decomposition stages. *Forests*, *12*(11). https://doi.org/10.3390/f12111473

**Table A2** Summary of species included in analyses. Plant growth form is specified for woody plants for trees (T) and shrubs (S), and for tree ferns (TF). The total number of replicates across all traits is reported for each species.

| Plant species | Plant family | Plant growth form | N |
| --- | --- | --- | --- |
| NATIVE |  |  |  |
| Acacia koa | Fabaceae | woody (T) | 18 |
| Alyxia oliviformis | Apocynaceae | woody (S) | 7 |
| Cheirodendron trigynum | Araliaceae | woody (T) | 4 |
| Cibotium glaucum | Cibotiaceae | fern (TF) | 10 |
| Dicranopteris linearis | Gleicheniaceae | fern | 46 |
| Diospyros sandwicensis | Ebenaceae | woody (T) | 7 |
| Diplazium sandwichianum | Athyriaceae | fern | 4 |
| Dodonaea viscosa | Sapindaceae | woody (S) | 16 |
| Dubautia menziesii | Asteraceae | woody (S) | 5 |
| Dubautia scabra | Asteraceae | woody (S) | 4 |
| Leptecophylla tameiameiae | Ericaceae | woody (S) | 10 |
| Metrosideros polymorpha | Myrtaceae | woody (T) | 336 |
| Osteomeles anthyllidifolia | Rosaceae | woody (S) | 8 |
| Pandanus tectorius | Pandanaceae | woody (T) | 7 |
| Psychotria hawaiiensis | Rubiaceae | woody (T) | 7 |
| Vaccinium calycinum | Ericaceae | woody (S) | 4 |
| Vaccinium reticulatum | Ericaceae | woody (S) | 4 |
| INVASIVE |  |  |  |
| Alstonia scholaris | Apocynaceae | woody (T) | 7 |
| Buddleja asiatica | Scrophulariaceae | woody (S) | 4 |
| Cecropia obtusifolia | Urticaceae | woody (T) | 7 |
| Chaetogastra herbacea | Melastomataceae | herb | 4 |
| Clidemia hirta | Melastomataceae | woody (S) | 11 |
| Cordyline fruticosa | Asparagaceae | geophyte | 7 |
| Elaphoglossum pteropus | Dryopteridaceae | fern | 4 |
| Eucalyptus saligna | Myrtaceae | woody (T) | 2 |
| Falcataria falcata | Fabaceae | woody (T) | 35 |
| Fraxinus uhdei | Oleaceae | woody (T) | 4 |
| Hedychium gardnerianum | Zingiberaceae | geophyte | 10 |
| Macaranga mappa | Euphorbiaceae | woody (T) | 7 |
| Mangifera indica | Anacardiaceae | woody (T) | 7 |
| Melastoma septemnervium | Melastomataceae | woody (T) | 7 |
| Melinis minutiflora | Poaceae | graminoid | 12 |
| Miconia calvescens | Melastomataceae | woody (T) | 4 |
| Myrica faya | Myricaceae | woody (T) | 4 |
| Passiflora tripartita | Passifloraceae | climbing | 4 |
| Prosopis pallida | Fabaceae | woody (T) | 2 |
| Psidium cattleyanum | Myrtaceae | woody (T) | 20 |
| Schizachyrium condensatum | Poaceae | graminoid | 8 |
| Setaria palmifolia | Poaceae | graminoid | 4 |
| Spathodea campanulata | Bignoniaceae | woody (T) | 4 |
| Sphaeropteris cooperi | Cyatheaceae | fern (TF) | 7 |
| Trema orientalis | Ulmaceae | woody (T) | 7 |

**Table A3** Total sample sizes for nine litter traits used in this study. Each replicate is a trait mean sampled from a species at a given site. There can be multiple replicates per species if they were sampled at multiple sites or by multiple studies.

| Trait | Sample size | |
| --- | --- | --- |
|  | Native | Invasive |
| C.mass | 24 | 27 |
| Ca.mass | 38 | 10 |
| CN.ratio | 37 | 28 |
| K.mass | 38 | 10 |
| lignin | 79 | 23 |
| LMA | 37 | 14 |
| Mg.mass | 38 | 10 |
| N.mass | 112 | 41 |
| P.mass | 92 | 29 |

**Table A4** Results from the tests for phylogenetic signal (Bloomberg’s K and Pagel’s λ) of litter traits, including both native and invasive species. Traits with significant phylogenetic signals are highlighted in bold font.

| **Traits** | **Bloomberg’s K** | | **Pagel’s λ** | |
| --- | --- | --- | --- | --- |
|  | ***K-value*** | ***P-value*** | ***λ-value*** | ***P-value*** |
| C.mass | 0.501 | 0.178 | 0 | 1 |
| Ca.mass | 0.704 | 0.530 | 0 | 1 |
| CN.ratio | 0.244 | 0.833 | 0 | 1 |
| K.mass | 0.864 | 0.166 | 1.239 | 0.056 |
| lignin | **0.458** | **0.035** | **1.025** | **0.049** |
| LMA | 0.251 | 0.404 | 0 | 1 |
| Mg.mass | 0.739 | 0.305 | 0.376 | 0.563 |
| N.mass | **0.507** | **0.002** | **0.999** | **0.001** |
| P.mass | 0.069 | 0.511 | 0.000 | 1.000 |

**Table A5** Litter trait data range for native and invasive plant species across all sites.

|  | **Unit** | **Plant origin** | **Min value** | **Max value** | **Range value** |
| --- | --- | --- | --- | --- | --- |
| C.mass | mg.g-1 | native | 302.8 | 520 | 217.2 |
| C.mass | mg.g-1 | invasive | 297 | 530 | 233 |
| CN.ratio |  | native | 22.9 | 179.7 | 156.8 |
| CN.ratio |  | invasive | 13.4 | 256.6 | 243.2 |
| Ca.mass | mg.g-1 | native | 5.1 | 31.36 | 26.26 |
| Ca.mass | mg.g-1 | invasive | 11.42 | 53.176 | 41.756 |
| K.mass | mg.g-1 | native | 0.3 | 3.945 | 3.645 |
| K.mass | mg.g-1 | invasive | 1.024 | 4.605 | 3.581 |
| LMA | g.m-2 | native | 44.05 | 395 | 350.95 |
| LMA | g.m-2 | invasive | 37.45 | 105.26 | 67.81 |
| Mg.mass | mg.g-1 | native | 0.5 | 5.8 | 5.3 |
| Mg.mass | mg.g-1 | invasive | 1.397 | 5.38 | 3.983 |
| N.mass | mg.g-1 | native | 1.2 | 23 | 21.8 |
| N.mass | mg.g-1 | invasive | 1.7 | 41 | 39.3 |
| P.mass | mg.g-1 | native | 0.03 | 0.8 | 0.77 |
| P.mass | mg.g-1 | invasive | 0.06 | 1.2 | 1.14 |
| lignin | mg.g-1 | native | 58 | 419.2 | 361.2 |
| lignin | mg.g-1 | invasive | 117 | 422 | 305 |

**Table A6** Results from the multivariate analyses of litter traits. Loading scores of the 9 litter traits from the principal component analysis (PCA) are reported.

| **Traits** | **PC1 (30.33%)** | **PC2 (25.60%)** |
| --- | --- | --- |
| C.mass | 0.15 | 0.48 |
| Ca.mass | -0.34 | -0.59 |
| CN.ratio | 0.5 | -0.21 |
| K.mass | -0.3 | -0.05 |
| lignin | 0.04 | 0.58 |
| LMA | 0.4 | -0.25 |
| Mg.mass | -0.48 | -0.47 |
| N.mass | -0.77 | 0.52 |
| P.mass | -0.66 | 0.36 |

**Table A7** Results from the multivariate analyses of climate across the study sites where litter trait data were sampled. Loading scores of the 9 climate variables and elevation from the principal component analysis (PCA) are reported.

| **Climate variables** | PC1 (55.33%)  high elevation, cool, dry to low elevation, warm, wet climates | PC2 (19.28%) warm and dry to cool and wet climates |
| --- | --- | --- |
| Annual cloud cover | 0.955 | 0.008 |
| Elevation | -0.627 | 0.342 |
| Annual humidity | 0.463 | 0.376 |
| Rainfall | 0.319 | 0.748 |
| Annual soil evaporation | -0.868 | -0.21 |
| Annual soil moisture | 0.599 | 0.712 |
| Annual solar radiation | -0.887 | -0.053 |
| Annual surface temperature | 0.773 | -0.601 |
| Annual temperature | 0.886 | -0.442 |
| Annual transpiration | 0.794 | -0.007 |

**Table A8** Summary results of the Mixed Random Effect models. Only traits with significant results are reported. Model 1 is to test for the effect of plant origin and its interactions with climate on litter traits when accounting for within-species variation, and Model 2 is to test if these effects shifted when controlling for the full effects of climate factors when accounting for within-species variation.

| **Traits** | **Variables** | **Est.** | **SE** | **df** | ***t-value*** | ***P-value*** |
| --- | --- | --- | --- | --- | --- | --- |
| Model 1: Trait ~ Plant.origin + Plant.origin:PC1 + Plant.origin:PC2 + (1\|Study) + (1\|Plant.species) | | | | | | |
| Lignin | Intercept | 5.214 | 0.1 | 39 | 51.83 | <0.001 |
|  | Plant.origin[native]:PCA2 | 0.93 | 0.02 | 76 | 4.53 | <0.001 |
|  | Plant.origin[invasive]:PCA2 | 0.009 | 0.068 | 69 | 0.13 | 0.897 |
| LMA | Intercept | 4.754 | 0.245 | 5 | 19 | <0.001 |
|  | Plant.origin[invasive] | -0.664 | 0.16 | 15 | -4.142 | <0.001 |
|  | Plant.origin[native]:PCA1 | -0.214 | 0.062 | 23 | -3.42 | 0.002 |
|  | Plant.origin[invasive]:PCA1 | -0.362 | 0.116 | 29 | -3.101 | 0.004 |
| CN ratio | Intercept | 4.199 | 0.148 | 33 | 28.286 | <0.001 |
|  | Plant.origin[invasive] | -0.379 | 0.155 | 31 | -2.48 | 0.018 |
| P.mass | Intercept | 0.472 | 0.047 | 38 | 9.917 | <0.001 |
|  | Plant.origin[native]:PCA2 | 0.016 | 0.011 | 90 | 1.439 | 0.153 |
|  | Plant.origin[invasive]:PCA2 | 0.203 | 0.059 | 92 | 3.43 | <0.001 |
| N.mass | Intercept | 1.775 | 0.129 | 49 | 13.675 | <0.001 |
|  | Plant.origin[invasive] | 0.361 | 0.163 | 47 | 2.215 | 0.031 |
|  | Plant.origin[native]:PCA2 | 0.057 | 0.033 | 126 | 1.7 | 0.091 |
|  | Plant.origin[invasive]:PCA2 ** | 0.208 | 0.088 | 132 | 2.369 | 0.019 |
| Model 2: Trait ~ PC1 + PC2 + Plant.origin + Plant.origin:PC1 + Plant.origin:PC2 + (1\|Study) + (1\|Plant.species) | | | | | | |
| Lignin | Intercept | 5.227 | 0.106 | 40 | 50.766 | <0.001 |
|  | Climate.PC2 | 0.082 | 0.02 | 77 | 3.98 | 0.002 |
| LMA | Intercept | 4.75 | 0.2 | 3 | 23.74 | <0.001 |
|  | Climate.PC1 | -0.155 | 0.056 | 6 | -2.735 | 0.034 |
|  | Plant.origin[invasive] | -0.564 | 0.149 | 17 | -3.77 | 0.001 |
| C:N ratio | Intercept | 4.097 | 0.16 | 25 | 25.49 | <0.001 |
|  | Plant.origin[invasive] | -0.386 | 0.152 | 31 | -2.533 | 0.016 |
| P.mass | Intercept | 0.405 | 0.066 | 33 | 6.05 | <0.001 |
|  | Plant.origin[invasive]:Climate.PC2 | 0.206 | 0.061 | 96 | 3.33 | 0.001 |
| N.mass | Intercept | 1.78 | 0.129 | 49.02 | 14 | <0.001 |
|  | Climate.PC1 | 0.012 | 0.024 | 65 | 0.515 | 0.608 |
|  | Plant.origin[invasive] | 0.358 | 0.167 | 47 | 2.188 | 0.033 |
|  | Plant.origin[native]:Climate.PC2 | 0.053 | 0.034 | 124 | 1.575 | 0.11 |
|  | Plant.origin[invasive]:Climate.PC2 | 0.207 | 0.088 | 129 | 2.349 | 0.02 |
| C.mass | Intercept | 6.063 | 0.044 | 9 | 126.68 | <0.001 |
|  | Climate.PC1 | -0.002 | 0.024 | 10.391 | -0.091 | 0.92 |
|  | Plant.origin[native]:Climate.PC1 | 0.049 | 0.014 | 26.231 | 3.51 | 0.001 |

**Table A9** Results of decomposition of variance analysis. Percentage of variance accounted by plant form, plant origin, among and within species for each litter trait.

| **Traits** | **Plant origin** | **Plant form** | **Among species** | **Within species** |
| --- | --- | --- | --- | --- |
| C.mass | 0 | 3.77 | 43.62 | 52.61 |
| Ca.mass | 0 | 0 | 28.53 | 71.47 |
| CN.ratio | 0 | 44.99 | 23.59 | 31.41 |
| K.mass | 4.04 | 0 | 50.84 | 45.12 |
| lignin | 0 | 1.12 | 55.01 | 43.87 |
| LMA | 41.7 | 18.55 | 22.93 | 16.82 |
| Mg.mass | 0 | 3.52 | 30.04 | 66.44 |
| N.mass | 0 | 23.47 | 28.33 | 48.19 |
| P.mass | 0 | 0 | 52.00 | 48.00 |

**Figure A1** Phylogenetic tree of 42 plant species used in this study. Black = native species, red = invasive species. ****

**Figure A2** Pearson’s correlation and variance inflation factor scores of the climatic variables.

The dashed red line represents a VIF threshold of ≥10, indicating significant multicollinearity among the climate variables.

**
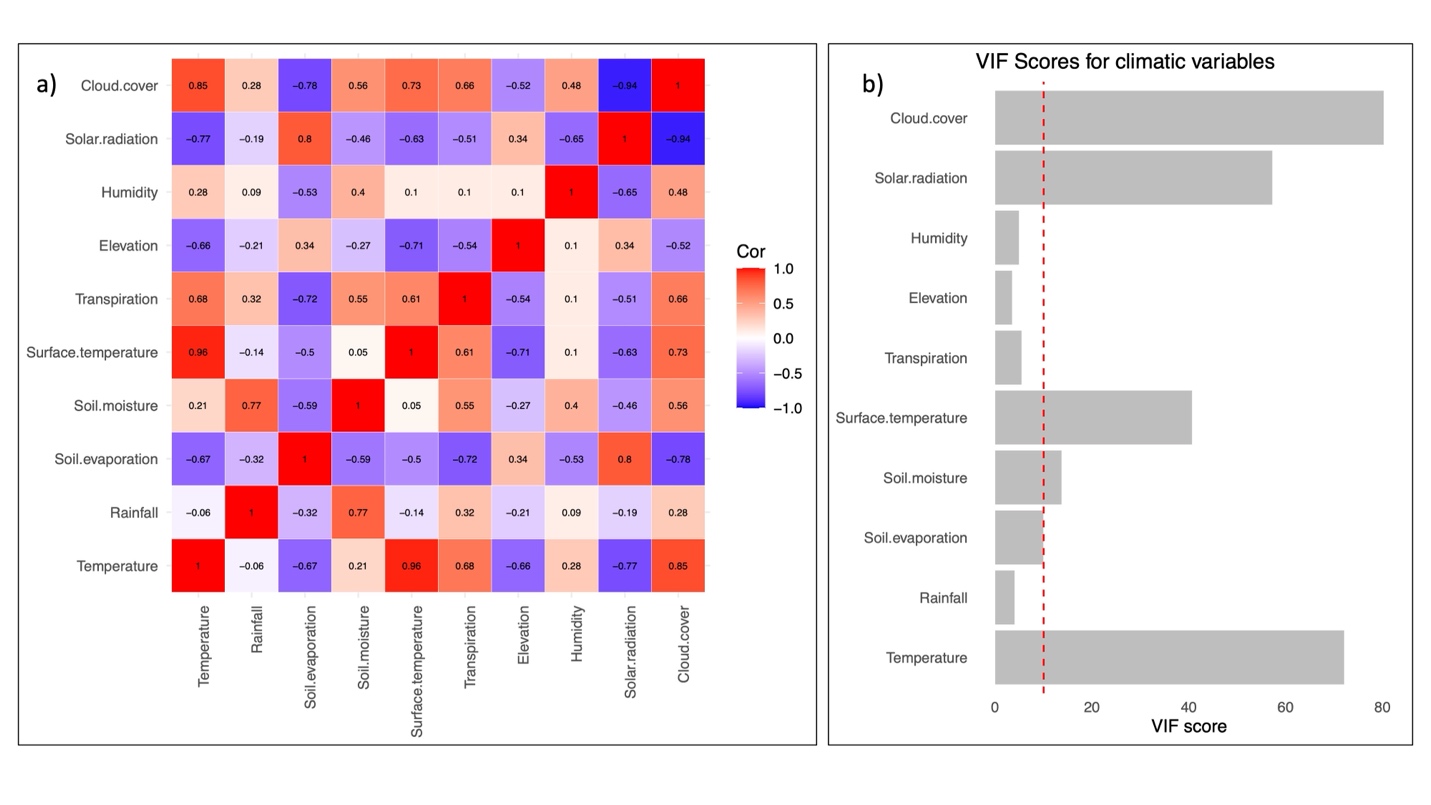
**

**Figure A3** Multivariate trait space of native and invasive plant species, using only data sampled on Hawaiʻi Island, characterized by nine key litter quality traits. The dominant native tree species ʻōhiʻa lehua (*M. polymorpha*) is also indicated due to its dominance in the dataset.

**Figure A4** Results of one-way ANOVA and Kernel density tests for trait data sampled only on Hawai‘i Island. a) summary of the raw litter traits data used in this study, * significant ANOVA test results comparing the mean litter trait values between native and invasive species at α < 0.05. b) Kernel density distributions of litter trait values comparing the endemic plant species ʻōhiʻa lehua (*M. polymorpha*), other native and invasive species, * the three distributions are statically significant at α < 0.05.

**Figure A5** Principal component analysis (PCA) plot of climatic variables used in this study.

**Figure A6** Relationship between litter traits and climatic factors, showing those traits with significant interactions between origin and climate. Growth form is designated for each species.
